# A critique of the alternating access transporter model of uniport glucose transport

**DOI:** 10.1007/s41048-018-0076-9

**Published:** 2018-11-16

**Authors:** Richard J. Naftalin

**Affiliations:** Physiology and Vascular Biology Group, King’s College London Medical School, Waterloo Campus, London, SE1 9HN UK

## Introduction

**Fig. 1:**
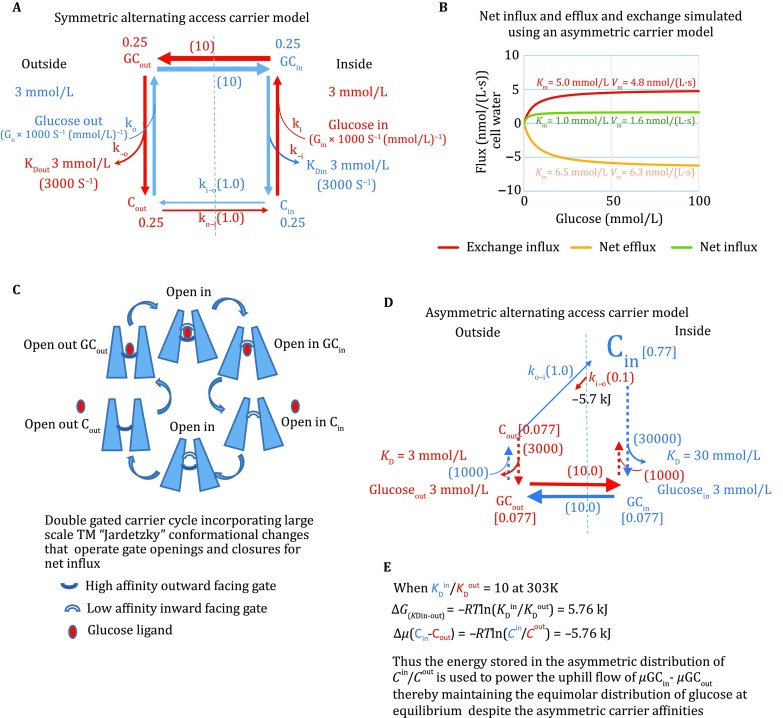
**A** Conventional representation of the symmetrical carrier model with the *K*_D_^in^ = *K*_D_^out^ = 3 mmol/L and *k*C_out–in_ = *k*C_in–out_. The *thicker arrows* represent higher flow rates of liganded carrier than those of the empty carrier. The *blue arrows* represent the influx pathway and the *red arrows* the efflux pathway. The symmetrical rates of ligand carrier transit *k*GC_out–in,_ *k*GC_out–in_ are 10× faster than the fast rate of empty carrier movement *k*C_out–in_, the second-order rates of ligand association with the external and internal carrier forms, *G*_out_*k*_out_ and *G*_in_*k*_in_ are assigned to be 1000× faster than *k*C_out–in_. **B** Conventional representation of the asymmetric alternating transporter model with parameters as illustrated in **D**. The simulation shows that *V*_m_ = 1.6 nmol/(L·s) for zero-*trans*- net influx with the parameters as in **D** is approximately 33% of the *V*_m_ for exchange uptake = 4.8 nmol/(L·s) and the *K*_m_ for net influx = 1.0 mmol/(L·s) is approximately 20% of the *K*_m_ for exchange influx = 5.0 mmol/L. The *V*_m_ for net efflux = 6.3 nmol/(L·s), *i.e.* 3.9× faster than net influx. **C** Jardetzky adaptation of gated asymmetric transporter. **D** Asymmetric single-cycle alternating carrier model. The lengths of the vertical lines represent the relative rates of association and dissociation. The relative lengths and widths of the horizontal lines represent the relative transit rates of loaded and unloaded carrier forms. The angular displacements of the horizontal rates represent the Gibbs free energy differences between the states. The free energy differences between liganded and unliganded states are not displayed. **E** Equations showing how asymmetric affinities of a single-cycle carrier enforce asymmetric rates of empty carrier distribution

The alternating access transporter model (AATM) was initially constructed to rationalize and explain a few readily quantifiable and reproducible experimental findings. The AATM, as originally conceived, consists of single specific binding site centrally situated within the cell membrane. The site alternately faces inwards and outwards. During the inversion process, the site can transport sugar across the membrane, and dissociation into the alternate bathing solution results in net transport. Return of the empty site reinitiates the transport cycle as illustrated in Fig. 1A. Glucose transport, which was demonstrated first in human red cells (Le Fevre and Le Fevre 1952) and sheep placenta (Widdas 1951), has high stereospecificity for D-pyranose sugars, like d-glucose and d-xylose in preference to l-sugars,* e.g.* l-fructose or non-transported l-glucose. This transport process has similarities to enzyme kinetics: both have saturation kinetics, the *K*_m_ being the concentration at which half maximal transport velocity, *V*_max_ obtained, is a measure of apparent affinity of ligand for the transporter (Fig. 1B). The process is passive in the human red cell, since at equilibrium the glucose concentrations in the extra and intracellular solutions are the same;* i.e.* net uphill accumulation does not occur.

An important kinetic finding, which implied the existence of mobile components within the passive glucose transporter, was the demonstration of countercurrent overshoot—uphill transport of isotopically labelled glucose into red cell cytosol, driven by a downhill counterflow of unlabelled sugar from the cytosol to the external solution (Thomas* et al*. 1957). The rationalization for this phenomenon was that the transporter behaved as a swing door exchanger, where the downhill drive of unlabelled glucose is closely coupled to an uphill flow of labelled sugar.

## Accelerated and equilibrium exchange and asymmetry

Equilibrium exchange experiments with equal concentrations of radio-isotopically,* e.g.* ^3^H- and ^14^C-labelled d-glucose, initially present, respectively, in the cytosol and extracellular solutions, show that the maximal rates of equilibrium exchange are much faster (≈10×) than of net influx and the *K*_m_ for the process also (≈10×) higher than the *K*_m_ for net glucose influx (Carruthers 1991; Carruthers* et al.* 2009). These asymmetric fluxes are represented according to the AATM in (Fig. 1A**).** Using several different protocols, it was shown that the glucose affinity on the export inside site of the erythrocyte transporter was approximately tenfold lower than for the glucose import site on the outside (Baker and Naftalin 1979; Carruthers* et al*. 2009; Karlish* et al*. 1972).

These findings were explained on the basis of adjustments to unidirectional rate constant of the symmetrical single-cycle carrier model (Fig. 1B, C), as originally envisaged (Baker and Widdas 1973; Geck 1971; Regen and Tarpley 1974).

A seductive feature of the alternating carrier model for glucose transport is that it rationalizes how accelerated exchange and counter flow of glucose may occur (Thomas* et al.* 1957; Wilbrandt and Rosenberg 1961). The explanation for accelerated ligand exchange is that the path for ligand exchange via the alternating carrier nodes inwards (C_out_ → GC_out_ → GC_in_ → C_in_) and outwards (C_in_ → GC_in_ → GC_out_ → C_out_) short-circuits the slow path of vacant-carrier outward transits (C_in_ → C_out_) or (C_out_ → C_in_) (Fig. 1A), where C_in_ and C_out_ represent the inwardly and outwardly facing empty carrier site and GC_in_ and GC_out_ the inwardly and outwardly facing liganded sites.

As well as having a higher rate, the glucose exchange process also has a higher *K*_m(exchange)_ than the *K*_m(net influx)_ (Fig. 1B). This difference between the *K*_m_s for net and exchange transport arises because both outside and inside sites must be fully saturated with ligand before exchange flux is maximal. Consequently, the *K*_m(equilibrium exchange)_ is mainly determined by the low-affinity inside site with a higher *K*_m(in)_, whereas the *K*_m(net influx)_ is determined only by the high-affinity external facing site (Baker and Widdas 1973; Naftalin 2008). The *K*_m_s for net flux are determined by monitoring the initial rates of net inward and outward flux with variable sugar concentrations in the *cis* bathing solution and nominally zero sugar in the *trans* solution. The concentrations giving half maximal velocities are defined as the *K*_m_s for zero-*trans* net influx or efflux. The *K*_m_ for equilibrium exchange is determined by monitoring the initial rates of unidirectional isotope flow when equal concentrations of labelled and unlabelled sugars are present in both inside and outside solutions. The *K*_m (equilibrium exchange)_ is again determined from the sugar concentration at which the unidirectional rate is half maximal.

The AATM postulates that when transporters alternate between the opposing sides of the membrane both the vacant carrier and ligand bound to the carrier site undergo spatial translation, or a phase translation that exposes them alternately to the inside and outside solutions. The ligand affinities are determined by apparent binding affinities on the opposite sides of the transporter with glucose concentrations in the external and internal aqueous solutions. If the opposing faces of the carrier have different affinities towards transported ligand, then the difference between Gibbs free energy of ligand at the binding sites is

1 $$ \Delta \Delta G_{{{\text{in}} - {\text{out}}}} = RT\ln \left( {{{K_{\text{D}}^{\text{in}} } \mathord{\left/ {\vphantom {{K_{\text{D}}^{\text{in}} } {K_{\text{D}}^{\text{out}} }}} \right. \kern-0pt} {K_{\text{D}}^{\text{out}} }}} \right), $$

where *K*_D_^*i*^ is the dissociation constant of ligand for the transporter site at any face *i* (Fig. 1E).

At equilibrium, when the rates of glucose exchange are equal, then to maintain detailed balance requires that the product of all clockwise rates within a single transport cycle equals the product of the anticlockwise rates (Boyd 1975).

When *K*_D_^in^ ≠ *K*_D_^out^, the distribution ratio of vacant sites and *C*^out^ and *C*^in^ at equilibrium is also forced to become unequal; hence,

2 $$ C^{\text{in}} /C^{\text{out}} = {{K_{\text{D}}^{\text{in}} } \mathord{\left/ {\vphantom {{K_{\text{D}}^{\text{in}} } {K_{\text{D}} }}} \right. \kern-0pt} {K_{\text{D}}}{\!}{\!}{\!}}^{\text{out}} . $$

3 $$ {\text{Since at equilibrium }}C^{\text{out}} k_{{{\text{o}}{-}{\text{i}}}} = C^{\text{in}} k_{{{\text{i}}{-}{\text{o}}}} , $$

where *k*_o−i_ and *k*_i−o_ are the unidirectional rates in inward and outward movement of unliganded carrier.

To obtain an asymmetric distribution of *C*^in^/*C*^out^ =  10 at equilibrium requires that *k*_o−i_/*k*_i−o_ = 10,

4 $$ \Delta G = - RT\ln \left( {{{k_{{{\text{o}}{-}{\text{i}}}} } \mathord{\left/ {\vphantom {{k_{{{\text{o}}{-}{\text{i}}}} } {k_{{{\text{i}}{-}{\text{o}}}} }}} \right. \kern-0pt} {k_{{{\text{i}}{-}{\text{o}}}} }}} \right). $$

Hence, at 30 °C when *k*_o−i_/*k*_i−o_ = 10 = −5.76 kJ/mol, where the activity of transported ligand (*L*^*i*^) is *L*^*i*^/*K*_D_^*i*^, superscript “*i*” refers to the internal or external solution concentration of transported ligand (glucose, mmol/L) (Fig. 1E).

Every asymmetric rate process requires energy to sustain the inequality in the opposing unidirectional rates (Naftalin 2008, 2010; Naftalin and De Felice 2012). An implicit assumption of the asymmetric AATM is that the energy stored in the asymmetric concentration distribution ratio of vacant carrier at equilibrium can be used to compensate for the energy difference due to the disparate affinities of ligands between the two sides. This brings the net energy change around the cycle to zero, and hence apparently no energy is expended in completion of the net transport cycle, as in Fig. 1A and D (Lapointe* et al.* 2009; Zhang and Han 2016a).

However, this assumption is unfounded because Gibbs’ phase rule imposes an additional constraint on phase equilibria, requiring that the chemical potentials of mobile components at equilibrium be equal in all phases to which they have access. Gibbs’ rule does not depend on any specific formulation concerning the constitution of matter (Mehra 1998). Thus, the implicit and erroneous assumption that the asymmetric distribution of unliganded carrier can be used as an energy source to compensate for the asymmetric affinities of the carrier in the internal and external phases—those parts of the transporter that are connected to the adjacent external solutions—is invalid.

Various arguments have been advanced in support of the legitimacy of the claim that asymmetric distribution of unliganded carrier is thermodynamically correct (Lapointe* et al*. 2009; Zhang and Han 2016a).

Given that the chemical potential of a substance B, *µ*_B_ in an ideal mixture is

5 $$ \mu_{\text{B}} = \mu^\circ_{\text{B}} + RT\ln \left( {x_{\text{B}} } \right), $$

where *µ*°_B_ is the standard free energy and *x*_B_ is the mole fraction of B.

In the non-ideal case this may be expressed as

6 $$ \mu_{\text{B}} = \mu^\circ_{\text{B}} + RT\ln \left( {a_{\text{B}} } \right), $$

where *a*_B_ is the activity of B and *a*_B_ = *x*_B_*γ*_B (_*γ*_B_ is the activity coefficient of B).

At equilibrium, since the activities and chemical potentials of a substance must be uniformly distributed between all connected phases i and j, it follows that

7 $$ a_{\text{B}}^{\text{i}} = x_{\text{B}}^{\text{i}} \gamma_{\text{B}}^{\text{i}} = a_{\text{B}}^{\text{j}} = x_{\text{B}}^{\text{j}} \cdot \gamma_{\text{B}}^{\text{j}} . $$

If at equilibrium *x*_B_^i^ ≠ *x*_B_^j^, it follows that since

8 $$ a_{\text{B}}^{\text{i}} = a_{\text{B}}^{\text{j}}, $$

then

9 $$x_{\text{B}}^{\text{i}} /x_{\text{B}}^{\text{j}} = \gamma_{\text{B}}^{\text{j}} /\gamma_{\text{B}}^{\text{i}} . $$

In the case of a mobile carrier, as the activities of all mobile components must be the same at equilibrium, then if *C*^in^/*C*^out^ = 10, then *γ*_C_^in^/*γ*_C_^out^ = 1/10.

It has been asserted that no energy difference is implied in the asymmetric distribution of carrier chemical potentials at equilibrium even though “the activities or concentrations or states 1 and 4 do not have to be equal” (Lapointe* et al.* 2007, 2009).

However, since at equilibrium the chemical potentials of all mobile components are equal, it follows that the asymmetric distribution of carrier component cannot be used as a driving force to offset the energy difference from the difference in binding affinities between the inside and outside sites. To prevent violation of Gibbs’ phase rule, any asymmetry in the distribution of empty carrier must be offset by a reciprocal asymmetric distribution of activity coefficients, and hence they would not provide any driving force to maintain the observed difference in affinities at the ligand binding sites.

This point has been tacitly conceded (Mueckler and Thorens 2013). Instead Mueckler and Thorens contend that the passive glucose transport system is symmetrical and its apparent asymmetries are due to the experimental error obtained owing to very rapid glucose fluxes at or above room temperatures. Zhang and Han (2016a, b) also claim that dissociation constants for glucose at the inside and outside of the transporter namely *K*_D_^in^ and *K*_D_^out^ are nearly identical.

Denial of the existence of transport asymmetry can be easily negated: GLUT1 has accelerated exchange kinetics; the *K*_m_ and *V*_max_ for exchange are much higher than for net influx at room temperature and the difference between the exchange and net flux parameters increases as temperature is reduced. The AATM can only rationalize the inequality in *K*_m_s between net and equilibrium exchange fluxes by assuming an asymmetric transporter, where the *K*_m(in)_ > *K*_m(out)_ (Brahm 1983; Cloherty* et al*. 1996; Naftalin and Rist 1994; Whitesell* et al*. 1989), so it is evident that GLUT1, when expressed in erythrocytes at least, does behave as an asymmetric transporter. The very wide differences in kinetic parameters between net influx and exchange flux, particularly in cold conditions ≈4 °C, provide incontrovertible support for the view that the differences between the kinetic parameters of exchange and net flux are real. Thus, any transport model must accommodate asymmetry and its implications regarding the transport mechanism.

Recently it has been suggested that the partition function *f*([S]), equivalent to ([C_out_] + [GC_out_])/([C_in_] + [GC_in_]), describes the ratio of total C_out_ and total C_in_ as a function of transported ligand concentration, [S]. This assumption implies that the sum of the carrier forms equilibrate between each side (Zhang and Han 2016a, b), rather than that both liganded and unliganded carrier separately equilibrate. This version of the conventional passive carrier model implies that binding energy of ligand with the carrier alters both the affinity of the binding sites and the unidirectional rates of transit of unliganded carrier and in the absence of transported ligand the empty carrier activity distribution will be uniformly spread between both sides. Then according to Zhang and Han’s partition distribution function, if the assigned unidirectional rates of liganded carrier movement across the membrane are faster than unliganded carrier, then as the inside site becomes progressively more saturated with ligand its affinity will be reduced and its mobility will increase relative to the external site.

Experimental results where the affinity of the inside site has been measured with low cytosolic glucose (Baker and Naftalin 1979; Cloherty* et al*. 1995) indicate that the affinity is approximately tenfold lower than that of the external site. These results do not support the prediction that affinity of the inside site for glucose decreases as the internal concentration rises.

A thermodynamic inconsistency with the assumption that the liganded and unliganded forms of carrier can be treated as equivalent indistinguishable mobile components (Zhang and Han 2016a) is that ligand binding is assumed to alter both the carrier affinity and mobility. This implies that the carrier consists of two differentiated mobile components. If the components have a differential mobility, they cannot be treated as being thermodynamically identical. If they do not have identical mobility, then they cannot be treated as a single thermodynamic component.

The asymmetry problem does not apply in the case of the insulin-sensitive glucose transporter GLUT4 which has repeatedly been shown to be symmetrical (Vollers and Carruthers 2012), nor does it have accelerated exchange transport, although it can be converted into a transporter with accelerated exchange by transferring helix 6 from GLUT1 into GLUT4.

A fundamental difference between enzyme and transport kinetics is that transporters incorporate steps involving transit of mobile ligand across a membrane between two adjacent membrane phases, whereas enzyme kinetics mainly describe chemical transformations within a single phase and in general do not require interphase transits of any component. So with enzyme kinetics the ligand concentrations seen as substrate and product are uniformly distributed within a single phase. This is not the case with transport kinetics, where transport across a membrane involves several phase changes. Hence, Gibbs’ phase rule must be applied to equilibrium states of transporters. The transport step,* i.e.* a translation between phases, is not the same as a chemical transformation within a single phase. The King–Altman diagrammatic approach (Peusner* et al*. 1985) adopted from enzyme kinetics, representing transport mechanisms as networks, treats chemical transformations from one chemical form to another and ligand translation from one phase to another as equivalent steps within a connected network (Kozuch 2015; Naftalin and De Felice 2012). (See Fig. 1A, D) This is not a legitimate assumption when considering asymmetric transport.

The conventional asymmetric alternating carrier model, as illustrated by the Jardetzky graphic cartoon (Jardetzky 1966), implicitly asserts that the high- and low-affinity forms of vacant carrier exist exclusively in one membrane phase and are absent from the other. This membrane-phase separation of ligand-bound transporter components ensures against ligand leakage across the transporter. However, according to Gibbs’ phase rule, at equilibrium any mobile component is uniformly distributed between all phases to which it has access. If the nominally “mobile” component does not have access to another phase, then it is not mobile and provides no connection between the phases. Equating the transporter with a rocker switch, as proposed for active transport (Jardetzky 1966), suggests that the transformation from high- to low-affinity glucose binding takes place midway between the membrane phases, during the transporter inversion process, so that there is a zero presence of the *cis* alternate mobile form in the *trans* phase. This implicit assumption ignores and sidesteps the necessity for isopotentials of all mobile forms in all phases to which they have access at equilibrium.

Gibbs’ phase rule when superimposed on the detailed balance requirement imposes an additional constraint on transport networks and prevents any passive form of asymmetric chemical potential distributions or rate processes of mobile components between phases. These dual constraints on interphase transport processes imply that unidirectional interphase steps must always be symmetrical and therefore cannot be regarded as similar to the association/dissociation steps or chemical transformation occurring in other parts of the King–Altman representations of the transport network where net energy flows are implied. This also invalidates the underlying assumptions of the asymmetric carrier model.

## Fixed asymmetric site model of transporters

**Fig. 2:**
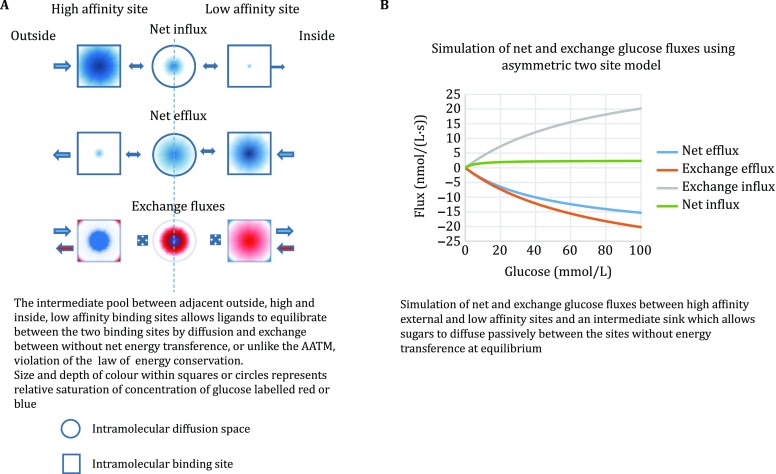
**A** Multisite model of glucose transport, the squares represent external and internal binding sites for net glucose influx and efflux. The inner site has a 10 × lower affinity 30 mmol/L than the outside site 3.0 mmol/L. The circles represent voids between binding sites through which glucose diffuses and equilibrates. During net efflux, the central void contains higher glucose concentrations than during net influx because the dissociation rate of glucose from the inside site is faster than the dissociation rate from the outside site. During equilibrium exchange, the void at the midpoint has similar amounts of both labelled sugars, so exchange is most favourable at the midpoint, although it is possible that there are other exchange sites. **B** Simulations of net glucose influx and efflux and equilibrium exchange flux with high-affinity external and low-affinity internal sites. An intermediate sink allows sugars to diffuse passively between the sites without net energy transference at equilibrium

The fundamental problems raised by the AATM can be averted by relinquishing the “mobile site” assumption and postulating instead that the transporter consists of an array of fixed ligand binding sites or nodes with differing,* i.e*. asymmetric affinities at the inside and outside surfaces. These nodes are connected via a channel which may have transiently open gates. Ligand flows occur by staged diffusion resulting from ligand dissociations and associations between the external solutions and vacant sites at the internal or external surface of the transporter and within the transmolecular pathway. Ligand diffusion occurs between adjacent sites within the network (Fig. 2A). Glucose, upon dissociation from a site, does not jump directly to the adjacent site. It must first diffuse via a separating segment of an intramolecular tunnel or cavity before it binds to the next node. The presence of intermediate sinks between each neighbouring binding sites requires that at equilibrium the ligand concentrations in these intermediate sinks will equilibrate with the concentrations in the external solutions. Thus, at equilibrium no net energy transference occurs from ligand transit between the high and low-affinity sites, because all the intermediate ligand source solutions will be at the same chemical potential as is present in the external solutions and on the binding sites.

The necessary presence of an intermediary ligand diffusion step avoids the thermodynamic and conceptual problems that AATM poses, where ligand transference between binding sites of unequal affinities implies energy transference, thereby necessitating a compensatory energy flow by a redistribution of vacant sites to retain detailed balancing. The core defect in the AATM lies in the assumption that ligand transport and chemical transformation of an enzyme–substrate are equivalent processes (Naftalin and De Felice 2012).

Thus, at equilibrium when the ligand concentrations are equal in both the external and internal solutions, there will also be a uniform ligand activity, or chemical potential at all nodes, whether or not they have the same affinity and also in the spaces between nodes. Thus, the entire ensemble will be in Detailed Balance. The model represented in Fig. 2A provides a theoretical basis within thermodynamic constraints for the main experimental findings relating to asymmetric net sugar fluxes. The lower panel illustrating exchange of glucose isotopes, blue and red, illustrates how a site capable of isotope exchange at the central binding site can explain exchange transport without requiring an alternative branch pathway.

## Accommodating the fixed site model of transport to accelerated exchange

Accelerated exchange requires that exchange of isotopic forms of ligands across the membrane transporter is faster than net flux. The fixed site transporter model (Fig. 2A, B) requires that exchanges between free ligand in solution and ligands bound to the transporter ligand binding sites are faster than net flux. More rapid exchange than net flow will occur between bound and free ligand, if the activation energy for exchange is lower than for the net dissociation. The lower activation energy for glucose exchange than net flux is well recognized (Abumrad* et al*. 1988; Brahm 1983). Conversely if the activation energy for exchange is higher than for net uptake and dissociation, then exchange retardation will occur, as has been observed with exchanges between different hexoses (Naftalin and Rist 1994; Cloherty* et al*. 1996).

The assumption that counterflow necessitates a mobile carrier was based on the belief that no other mechanism for exchange existed (Thomas* et al*. 1957). However, faster isotope exchanges than net dissociative flux have commonly been observed in chemical and biochemical catalysis. An example where rapid exchange occurs at fixed binding sites is deuterium–hydrogen exchange on nickel catalysts,* e.g.* hydrogen production and deuterium-proton exchange reactions catalysed by platinum or tungsten (Bonheoffer and Farkas 1932; Eley 1951). Thus, bound ligand can dissociate from the catalytic site, either by rapid exchange with adjacent ligand or by a net dissociation of ligand from the primary hydration shell. These conditions are easily accommodated within the fixed site network models shown in Fig. 2A with sites where more than one ligand can be in close proximity to the binding site Carruthers* et al*. 2009; Cunningham and Naftalin 2013, 2014; Naftalin 2008.

## Interpreting transporter structure and function from crystallographic structures of gluts

Recent papers (Lin* et al*. 2012; Quistgaard* et al*. 2013, 2016) reveal eight separate XylE conformers, five open inward, two holo (PDB 4JA3) and three apo forms (PDB 4JA4) and three open outward holo conformers (PDB 4GBY, 4GBZ and 4GCO). These eight XylE structures have been used as templates to build a homology-based model of GLUT1 incorporating the structured endofacial linkers. The human GLUT1 crystal structure has more recently been resolved in the inward open conformation, and Deng *et al*. (2014) found it to be similar to XylE (PDB 4PYP). Additionally, the crystallographic structures of GLUT3 at high resolution, 1.5 Ǻ in the outward occluded conformation (PDB 4ZW9, 4ZWB, 4ZWC) have been published (Deng* et al*. 2015).

These key crystallographic findings affirm some of the predictions of the rocker switch model of uniport transport (Deng* et al*. 2015; Quistgaard* et al*. 2016) (Table 1). The main features observed are a centrally located high-affinity binding site for glucose and glucose derivatives lying within a centrally located cleft between the six N- and six C-terminal TMs. Additionally, the observed open outward, open inward and occluded conformer poses of XylE satisfy most of the criteria required of the rocker switch alternating access mode of uniport transport for d-glucose.

**Table 1:** Comparison of the properties of the symmetrical, asymmetrical and multisite models for glucose transport

Condition	AATM asymmetric free carrier rates	AATM symmetrical carrier	Branched multisite model
Asymmetric *K*_m_ influx < efflux	✓	×	✓
Asymmetrical *V*_max_ influx < efflux	✓	×	✓
*V*_max_ exchange influx > *V*_max_ net influx	✓	×	✓
*K*_m__exchange influx_ > *K*_m__net influx_	✓	×	✓
Haldane ratio for net fluxes (*V*_max in–out_/*K*_m in_)/(*V*_m out-in_/*K*_m out_)=1	×	×	✓
GLUT1DS T295M inhibition of *V*_max_ net influx no change in *K*_m_ influx	✓	✓	✓
GLUT1DS T295M inhibition of *V*_max_ net efflux decrease in *K*_m_ efflux	×	×	✓
Crystallographic docking showing high-affinity central site	✓	✓	✓
In silico docking showing multiple low-affinity docking sites	×	×	✓
Inward and outward facing conformers	✓	✓	✓
GLUT1DS with absent docking from M295 site	×	×	✓
Transport without conformer inversion	×	×	✓
Single unbranched cyclic uniporter network model	✓	✓	×
Branched multi cycle network	×	×	✓

Molecular dynamics simulation studies demonstrate that the GLUT1 six N-terminal transmembrane (TM) helix N-domain is relatively immobile in comparison with the six TM C-terminal domain which contains segments that bend inwards and outwards during inward–outward facing transition as a result of discontinuities in TM7 and TM10 (Fu* et al*. 2016b; Park 2015; Quistgaard* et al*. 2016). Additionally, the intracellular structured domains of the 6–7 linker region also undergo some conformational shifts in association with the C-terminal domain. These studies confirm that there are low-affinity glucose binding sites within the external and internal vestibules, previously identified using docking methods on the static templated GLUT1 structures (Cunningham* et al*. 2006; Cunningham and Naftalin 2013, 2014). The low-affinity external sites exposed to the external solution may act as ligand sensors and attractants.

## Glucose transporter deficiency disorder GLUT1DS

There are numerous genetic disorders affecting the glucose transporter GLUT1. These GLUT1 deficiencies give rise to a syndrome characterized by epileptic seizures in infants that do not respond to normal anti-epileptic drugs and can be initiated by fasting or exercise (Leen* et al*. 2010). The GLUT1DS mutation T295M is sited in the external linker between TM7 and 8, close to the rim of external vestibule. It inhibits glucose transport kinetics by reducing both *V*_max_ and *K*_m_ of net glucose exit whilst affecting the influx parameters to a lesser extent, by approximately 50% (Cunningham and Naftalin 2013; Wang* et al.* 2003). Thus, the mutation greatly alters the transporter kinetic asymmetry. In control erythrocytes, the affinity ratio for d-glucose *K*_m(in)_/*K*_m(out)_ ≈10 is asymmetric and with the T295M mutation the *K*_m(in)_/*K*_m(out)_ ≈0.6 (Table 1).

Our in silico docking studies have shown that one of the external glucose binding sites on GLUT1 is occluded by the mutation T295M. Docking shows that some of the rotamer postures of the GLUT1 M295 mutated methionine side chain prevent ligand binding and passage via one of the available two tunnel openings between the external solution and external vestibule (Fujii* et al*. 2007, 2011; Wang* et al*. 2008).

The mutation is ≅20-Ǻ distant from the central binding site, where the main high-affinity binding and alternating action is assumed to occur. This poses the question as to how a mutation at the transporter's external surface can alter the apparent affinity of the inward facing transporter site without significantly affecting external affinity. These mutation-induced kinetic changes undermine the central assumptions on which alternating access model of glucose transport is based as they show that a change at the external surface of the transporter can radically alter the transport symmetry which the AATM contends is mainly controlled by the relative rates of inward and outward flows of the empty carrier that is supposed to reside in the centrally situated binding site.

The basis of the AATM is that the rate constants of the network represent unidirectional rates of flow of either unloaded C or loaded CG carrier forms within the central single site ligand docking region. Yet it is evident that a T295M mutation at the exofacial margin of the transporter has a dramatic effect on the asymmetric glucose transport parameters, changing the Haldane ratio, namely (*V*_max(out)_/*K*_m(out)_)/(*V*_max(in)_/*K*_m(in)_) from ≈1 in control to ≈0.3 (Table 1). Since one of the requirements of the cyclic network model of the AATM is that the Haldane ratio should always be close to 1 (Helgerson and Carruthers 1987; Krupka 1989), the alteration in the ratio by the T295M mutation and several others (Wong* et al*. 2007) shows that the simple view that glucose transport kinetics can be adequately described on the basis of a single reversible cycle is inconsistent with the kinetic data as well as the thermodynamics.

**Fig. 3:**
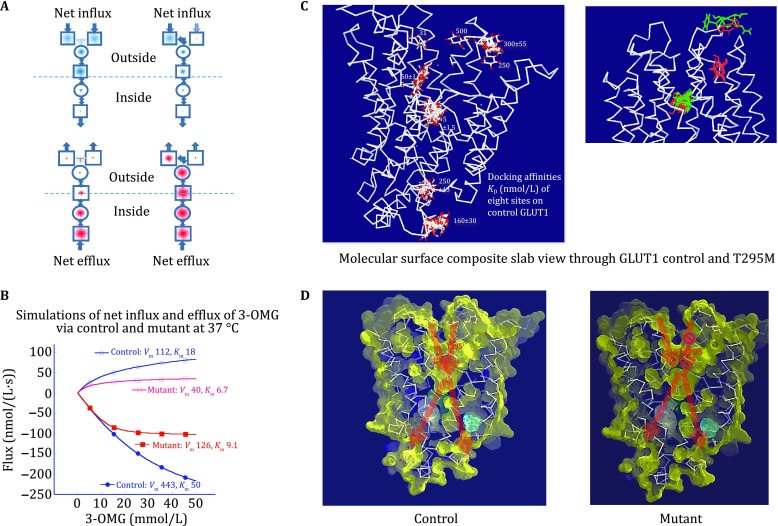
**A** Branched network model of glucose transport illustrating the effects of the T295M mutation on net influx and efflux at 37 °C. The square and circle symbols represent the same as in Fig. 2A. The right branch to the external vestibule is blocked by the M295 mutation in GLUT1DS and reduces net influx by 50% but without greatly affecting *K*_m_ net influx. However, the M295 mutation has a much larger effect on net efflux as slowing efflux leads to an accumulation of glucose within the external vestibule with a tailback that retarded glucose efflux and a reduces the *K*_m_ net efflux. **B** Simulations of the comparative effects of control and mutant glucose net influx and efflux (Cunningham and Naftalin 2013). **C** Docking studies of GLUT1 showing docking positions of glucose. The effect of the T295M mutation on glucose docking is illustrated in top right panel where the green stick model of glucose is absent from the mutant at the external vestibular tunnel but present in control (red stick) glucose. **D** Comparison of tunnels in control and M295 mutants. The tunnel between the external solution and vestibule is occluded by the mutant

An explanation for the effect of GLUT1DS T295M mutation based on the structural information on GLUT1 available from crystallography is that it blocks one of the two tunnels between the external solution and external vestibule (Fig. 3A–D). This induces an asymmetric effect on glucose transport kinetics in the following way: the rate limiting step to net glucose uptake is via the narrow bottleneck at the central binding site where, as is now demonstrated by MD simulation, glucose has to undergo a 180° rotation to negotiate its way around a chicane (Fu* et al*. 2016b; Martens* et al*. 2016; Sun* et al*. 2012). The T295M GLUT1 DS mutation blocks one of the two tunnel openings between the external solution and external vestibule (Fig. 3A, C). Thus, in the zero-*trans* net exit condition, when the cytosol is pre-loaded with high glucose concentrations, blocking one of the possible exits from the vestibule into the external solution results in glucose accumulation within the vestibule. This will cause a tailback of sugar ligands in the transport channel and hence slow glucose efflux and also reduce the *K*_m_ of net exit (Fig. 3A, B). During net influx, the T295M mutation will slow net glucose entry and therefore slow its concentration build-up within the external vestibule due to the bottleneck to glucose flow at the central site. Hence, the T295M mutation will have a relatively smaller retarding effect on uptake than on exit. This provides an explanation for large reversal of transporter kinetic asymmetry induced by the T295M mutation. For further details see the reference Cunningham and Naftalin 2013.

This relatively straightforward explanation for asymmetric glucose transport both in the control and mutated condition supports the view that glucose transport is a form of staged diffusion along the branched central channel (Cunningham and Naftalin 2014) and is summarized in Table 1.

## Docking and molecular dynamics in silico methods

The multiple conformers and isomeric structures of XylE and GLUTs have been used to construct an alternative transport trajectory and mechanism whose predictions deviate from the large-scale coordinated rigid-body conformational changes in TMs that the transporter is expected to undergo in alternating access modes (Cunningham and Naftalin 2013, 2014; Deng* et al*. 2015; Fu* et al*. 2016b; Park 2015).

Many large and small intramolecular voids or cavities exist within GLUTs, the largest of these are the external and internal vestibules, termed tunnels, as they are accessible to the external or internal bathing solutions, respectively. The vestibules have variable volumes, dependent on whether the transporter is in the inward or outward facing conformation, or filled with bound ligands (Cunningham and Naftalin 2014, Fig. 4A, B). Intramolecular cavities of variable size are also present depending on the conformer conformation and external forces exerted on the transporter by the membrane (Iglesias-Fernandez* et al*. 2017). These cavities are inaccessible to the external surface. The majority lie within the central cleft between the N and C domains and also serve as sugar docking sites. It is evident from superimposition of all eight XylE conformers that interchange between the tunnel and cavity forms generates a potential channel between the external and internal surfaces via the central cleft. The interchanges between conformers will lead to transient opening of gates between the external vestibule and central cavity. Thus, potentially both water and sugar ligands may cross the entire channel by a staged diffusion process as already envisaged for water (Li* et al*. 2013) and corroborated by several other MD studies of GLUTs (Deng* et al*. 2014, 2015; Deng and Yan 2016; Fu* et al*. 2016). The staged diffusion will be partially controlled by the gating rates,* i.e.* the rates of bulky side chain rotamer interchanges that form the channel bottlenecks.

**Fig. 4:**
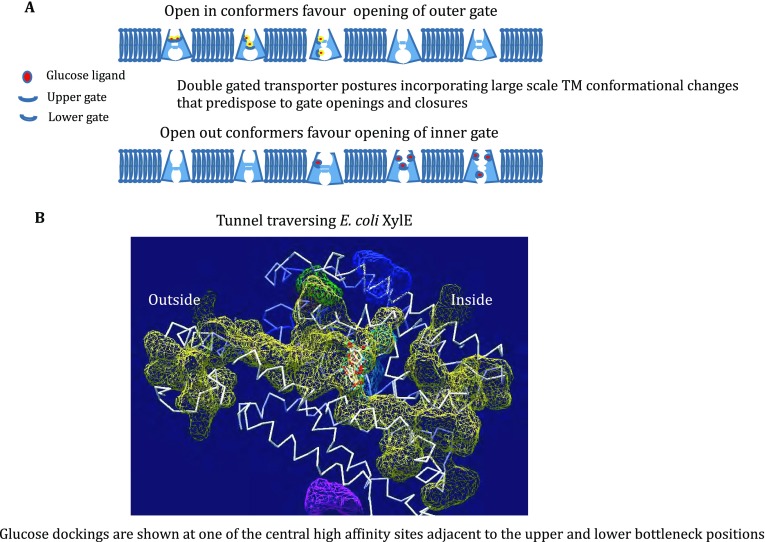
**A** Graphic simulating various phases of the staged diffusion model of glucose transport via GLUT1 where gates operated by small scale conformation change permit net and exchange transference across the transport network. **B** Figure showing the central tunnel traversing XylE isoform 4GC0 (Quistgaard* et al*. 2013; Cunningham and Naftalin 2014). The yellow mesh shows the tunnel limits as determined by the program

This scenario has been partially confirmed recently by atomistic molecular dynamic demonstrations that the GLUT1 central channel contains bottlenecks that act as gates to both water and ligand flow. The gate opening probabilities are partial functions of lateral forces exerted by the lipid bilayer, which has a temperature-sensitive dynamic structure, as well as the forces exerted by the internal elastic properties of the transporter molecule (Iglesias-Fernandez* et al*. 2017).

Temperature-dependent fluid to gel phase transformations result in abrupt slowing of glucose transport via the glucose transporter GLUT1 (Tefft* et al*. 1986). Molecular dynamic simulation of membrane-phase changes from fluid to gel states demonstrate increases the bilayer thickness by straightening apposing lipid chains. The lateral forces exerted by the membrane on the transporter squeeze the central part of the transporter and elongate its *Z*-axis. This decreases the volume of the cavities in the central channel and displaces the contained water molecules to the external and internal vestibules. Selective thermostatic controls on membrane and proteins show that these effects are mainly exerted by the membrane forces irrespective of the transporter temperature. These simulations support the view that at least one mode of transmembrane glucose transport is via staged diffusion without necessitating large-scale conformational changes in the protein.

## Evidence supporting transporter inversion as an optional transport mode

Advances in atomistic molecular dynamic simulations have now shown that the plant disaccharide transporter SWEET (Sugar Will Eventually be Exported Transporters) containing seven transmembrane helices can adopt conformations that match the open in, occluded and open out conformers demonstrated by crystal structures (Han* et al*. 2017; Latorraca* et al*. 2017). The relatively small size of the SWEET transporter coupled with very advanced high-performance computations allows atomistic demonstrations of spontaneous unbiased large-scale inversions, (1 transition per virtual 14 µs of simulation). Interestingly, Latorraca* et al*. suggested that substrate transits the membrane transporter, whilst the transporter adopts the same conformations and undergoes the same transitions in the presence and absence of the substrate. Glucose in the SWEET transporter can move independently of the transporter in the *z* plane of the transporter, and rotate in the *x*–*y* plane within the transporter cavity.

That ligands “take a free ride” through the semiSWEET transporter implies that they do not alter the kinetics of inversion by interaction with the native conformational states of the unliganded transporter. However, another study using single-molecule Forster resonance energy transfer (smFRET) in A*rabidopsis thaliana* (AtSWEET13) suggests that substrate binding altered the number of FRET states from two in the apo state to one, stabilizing the holo bound conformation in an inward facing state (Han* et al*. 2017). These latter studies suggest that these conformational changes may be consistent with formation of a dimer structure where the adjacent structures act in concert, as has been suggested already for GLUTs (Carruthers* et al*. 2009).

## Conclusions

These results with SWEET transporters may not fully reflect the conformational changes that occur in GLUTs which are much larger and have more complex extramembranous structures, particularly in the cytosolic domains. Much remains to be demonstrated with atomistic and perhaps also coarse-grained Martini MD simulations of sugar transport before an informed view regarding allosteric or induced-fit interactions of the transporter with transported and non-transported inhibitory ligands can be assigned with confidence. Nevertheless, the observed kinetics and thermodynamics of glucose transport via GLUT1 are clear enough to show that they are inconsistent with the AATM and thus some form of staged diffusion is currently a more satisfactory description of both control GLUT1 transport and for several GLUT1DS mutations.
